# Norway rats recruit cooperation partners based on previous receipt of help while disregarding kinship

**DOI:** 10.1016/j.isci.2024.111314

**Published:** 2024-11-04

**Authors:** Sacha C. Engelhardt, Niklas I. Paulsson, Michael Taborsky

**Affiliations:** 1 Department of Sociobiology and Anthropology, Johann-Friedrich-Blumenbach Institute for Zoology and Anthropology, University of Göttingen, 37077 Göttingen, Lower Saxony, Germany; 2 Behavioural Ecology and Sociobiology Unit, German Primate Center, Leibniz Institute for Primate Research, 37077 Göttingen, Lower Saxony, Germany; 3 Behavioural Ecology, Institute of Ecology and Evolution, University of Bern, 3032 Hinterkappelen, Bern, Switzerland; 4 Department of Collective Behavior, Max Planck Institute of Animal Behavior, 78467 Konstanz, Baden-Württemberg, Germany

**Keywords:** Rodent behavior, Zoology, Evolutionary biology

## Abstract

Norway rats are known to liberate trapped conspecifics, which implies an empathic response to the deplorable situation of the captive. If this is an altruistic behavior reflecting an evolved decision rule, the requisite fitness enhancement to the actor may result either from close relatedness or the expectation of future returns. Neither potential effects of relatedness nor of reciprocal returns have yet been examined. Our two-stage experiment revealed that wild-type Norway rats preferably collaborated with partners that had previously freed them from a trap and subsequently cooperated with each other, indicating that expected future benefits may underlie the deliverance of trapped companions. Relatedness had no effect on their cooperative propensity. These results show that rats recruit partners to coordinate cooperation by direct reciprocity but not kin discrimination, suggesting that the evolutionary mechanism responsible for the altruistic liberation behavior of Norway rats may be reciprocal altruism rather than kin selection.

## Highlights

• Social partners released or did not release focal rats from a confinement tube
• Subsequently focal rats cooperated more with previous liberators
• Focal rats coordinated according to the direct reciprocity decision rule
• Relatedness had no effect on the coordinated cooperation of focal rats

## Graphical abstract

**Figure:**
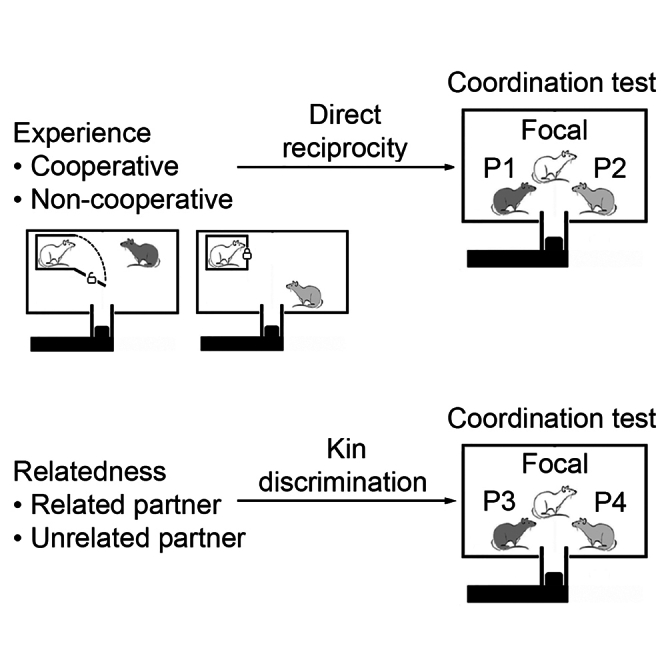

## Introduction

Some animals free trapped conspecifics,^1^^,^^2^^,^^3^^,^^4^^,^^5^^,^^6^^,^^7^^,^^8^^,^^9^^,^^10^^,^^11^^,^^12^^,^^13^^,^^14^ which has often been interpreted as indication of empathy.^15^^,^^16^^,^^17^^,^^18^^,^^19^^,^^20^ Even if the role of empathy in this prosocial helping behavior has been contested,^21^^,^^22^^,^^23^^,^^24^^,^^25^^,^^26^^,^^27^^,^^28^^,^^29^^,^^30^ it seems possible that such seemingly altruistic acts reflect an evolved adaptive response to a deplorable situation of a social partner. Nevertheless, apart from information on the significance of familiarity and relatedness for the rescue behavior of ants,^5^^,^^31^ it is currently unclear which selection mechanism might cause the emergence of liberation behavior in animals. In principle, such prosocial helping behavior may evolve if it raises the inclusive fitness of the actor due to relatedness with the victim or because of increased chances to receive future returns, in other words either by kin selection or reciprocal altruism.^32^^,^^33^

In various taxa it has been shown that social partners are able to coordinate actions to collectively fulfill a task, including cichlid fishes,^34^ parrots,^35^^,^^36^ corvids,^37^^,^^38^ elephants,^39^ rats,^40^ wolves,^41^ domestic dogs,^42^^,^^43^ dolphins,^44^ and primates.^45^^,^^46^ It is often unclear which social information partners of experimental dyads are using when accomplishing such coordinated cooperation. It might be expected that the quality of a potential cooperation partner derived from its previous behavior provides an important clue on its cooperative propensities. Homophily, i.e., similarity of non-social personality traits and heterophily, i.e., dissimilarity of social personality traits between partners were found to affect partner choice in macaques and were positively associated with cooperation in a coordination task.^47^^,^^48^ Moreover, chimpanzees were found to prefer recruiting known cooperative partners for help in a coordination task,^49^ and several species, e.g., ravens, chimpanzees, bonobos, hyenas and keas, were shown to choose cooperation partners dependent on their relationship quality.^50^^,^^51^^,^^52^^,^^53^^,^^54^

Norway rats (*Rattus norvegicus*) are highly social animals that nest in large colonies.^55^^,^^56^ They can distinguish between kin and non-kin,^57^^,^^58^^,^^59^ between different degrees of relatedness,^60^ and between single individuals, i.e., showing true individual recognition.^61^ Experimental studies showed that Norway rats give more and earlier help to cooperative partners than to non-cooperative partners.^62^^,^^63^^,^^64^^,^^65^^,^^66^^,^^67^^,^^68^^,^^69^ Male Norway rats cooperated by applying the direct reciprocity decision rule, whereas kinship reduced their propensity to return received help.^59^ Female Norway rats were shown to help according to the quality of help they received^67^^,^^70^ and their partner’s need.^71^^,^^72^^,^^73^ Studies have revealed that Norway rats are capable of comprehending the need for, and role of, others in various tasks and games which require at least two individuals to complete.^40^^,^^62^^,^^74^^,^^75^

**Figure 1:**
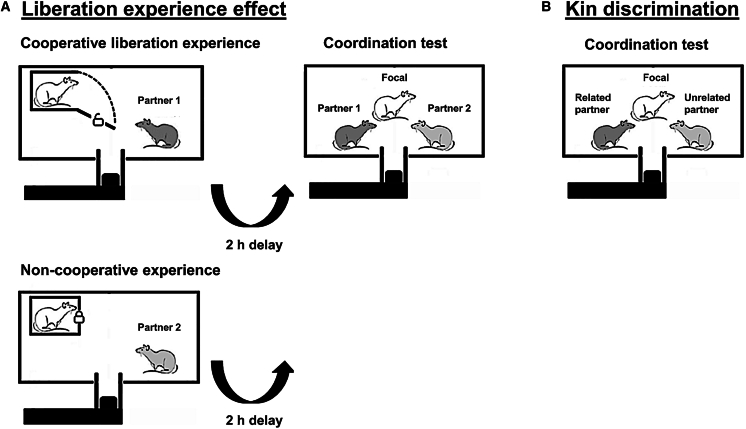
Experimental design to study coordinated cooperation The left side (A) illustrates the cooperative and non-cooperative experience phases in the direct reciprocity decision rule experiment. Focal rats experienced these two experience phases in a random order, and the focal rat was placed inside a transparent and perforated confinement tube. In the non-cooperative experience phase, the non-cooperative partner outside the tube was incapable of opening the confinement tube’s door to free the focal rat, but it could move the food-baited platform to access food rewards for itself. In the cooperative experience phase, the partner rat could open the confinement tube’s door thereby freeing the focal rat, and only thereafter the food-baited tray could be moved by both rats pulling the sticks together to access the food rewards. There was a 2-h break between the two expereince phases and a 3-h delay between the second experience phase and the test phase. In the test phase, the focal rat was placed freely in the test cage together with the two previous partners, one cooperative and one non-cooperative. The right side (B) illustrates the test for the kin discrimination experiment which did not involve an experience phase; in this experiment we used a related (full sister) partner and an unrelated (not a full sister) partner. The focal rat and both partners were cage mates, therefore both partners were similarly familiar to the focal subject.

As collective behaviors such as coordinated food acquisition might well reward all involved parties, it is important for the study of cooperation to understand which evolutionary stable decision rules are used to determine which partners are preferentially recruited to participate in the operation. In this study, we tested for prosocial behavior according to two possibilities, kin selection or reciprocal altruism, by using wild-type Norway rats with a two-staged experimental design, which involves liberation from a trap tube, coordination in food acquisition, and a combination of either related or unrelated experimental dyads. We used a coordination task to test whether previously received help affects the propensity of rats to cooperatively acquire a food reward. We further asked whether this would also affect their success rate in a physically challenging operation. We hypothesize that rats will show a preference for partners based on correlated payoffs,^33^^,^^76^ either through (1) past cooperative experience (direct reciprocity^77^), or through (2) kinship.^78^^,^^79^ To assess if focal rats coordinate cooperation according to the direct reciprocity decision rule, we predicted that focal rats should (1) coordinate more often, (2) have shorter latencies to the first coordinated action, and (3) have shorter intervals between coordinated actions with a cooperative partner, who previously freed them from a confinement tube to allow for coordinated cooperation, than with a non-cooperative partner, who did not previously free them from a confinement tube (Figure 1A). Several past studies have trained rats to free partners from confinement,^2^^,^^22^^,^^24^^,^^80^^,^^81^^,^^82^ and the social contact from a freed partner has been shown to be rewarding to them^22^^,^^24^^,^^80^^,^^81^^,^^82^ without being distressing to the occupant after habituation.^25^ We use this social reward of being freed by a partner as an incentive to recruit this partner to solve a coordinated pulling task for fetching a food reward. To assess if focal rats coordinate cooperation according to kin discrimination, we predicted that focal rats should (1) coordinate more often, (2) have shorter latencies to the first coordinated action, and (iii) have shorter intervals between coordinated actions with a related partner than with an unrelated partner (Figure 1B).

## Results

### Previous help experience and coordinated cooperation

**Figure 2:**
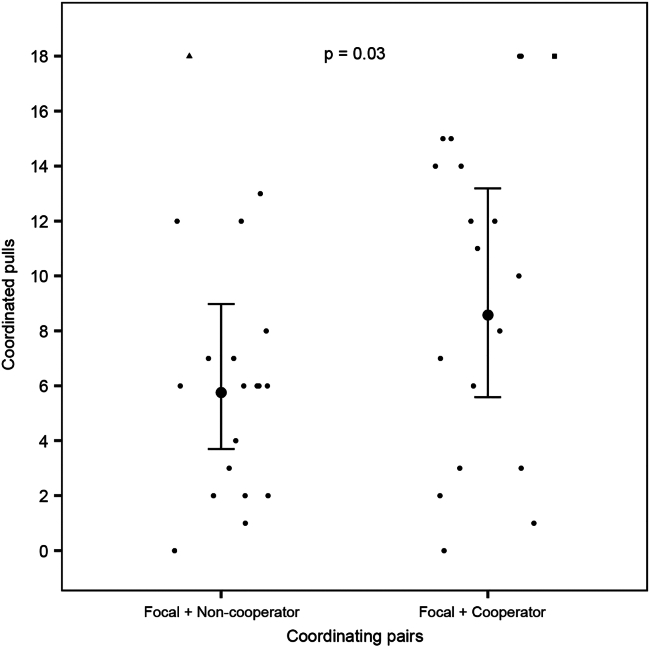
The direct reciprocity decision rule and coordinated pulling The number of coordinated pulls in the test phase performed by the coordinating pairs, i.e., focal rats with previously cooperative partners vs. focal rats with previously non-cooperative partners. The large dots with the whiskers represent the mean number of coordinated pulls with 95% confidence intervals, respectively, and the dots represent the raw pulling data. The black square and the black triangle points at the top of the graph represent values of 24 and 32 coordinated pulls, respectively, and are graphed here at 18 coordinated pulls, where the ordinate was truncated to enhance resolution. The *p* value for the comparison is equal to 0.03.

In the test phase, focal rats with partners that had liberated them beforehand from a captive tube coordinated joint pulls of a tray baited with food more often (48% more coordinated pulls) than focal rats with non-cooperative partners that had not released them from the tube (Estimate ±SE: 0.40 ± 0.18, *p* = 0.03, Figure 2). The comparison between the full model and the null model without previous experience revealed that previous cooperative experience significantly affected the number of coordinated pulls (Χ^2^ = 3.95, *p* = 0.047). These results supported our prediction of the direct reciprocity decision rule for coordinated cooperation. Overall, the number of coordinated pulls in the test phase was not influenced by the number of coordinated pulls between the focal rats and the partners in the experience phase (Estimate ±SE: 0.001 ± 0.007, *p* = 0.87), the mass difference between the focal rats and the partners (Estimate ±SE: −0.0001 ± 0.0033, *p* = 0.97), and the sequence of experience phases (Estimate ±SE: −0.29 ± 0.27, *p* = 0.28). Previous experience with pulling training (Estimate ±SE: 0.19 ± 0.36, *p* = 0.60) and previous experience with door-opening training (Estimate ±SE: −0.39 ± 0.40, *p* = 0.33) did not influence the number of coordinated pulls in the test phase.

The number of coordinated pulls in the test phase between focal rats and the cooperative partners only was not influenced by the number of coordinated pulls in the experience phase between the focal rats and the cooperative partners (Estimate ±SE: 0.004 ± 0.011, *p* = 0.74)), the mass difference between the focal rats and the cooperative partners (Estimate ±SE: 0.002 ± 0.004, *p* = 0.67), the sequence of experience phases (Estimate ±SE: −0.30 ± 0.23, *p* = 0.19), previous experience with pulling training (Estimate ±SE: 0.16 ± 0.36, *p* = 0.66) and previous experience with door-opening training (Estimate ±SE: −0.50 ± 0.52, *p* = 0.34). There was no difference between the full model and the null model without the number of coordinated pulls between the focal rats and the cooperative partners in the experience phase (Χ^2^ = 0.11, *p* = 0.74). The latency until the trapped focal rats became free from the captive tube was not affected by the experience phase (cooperative vs. non-cooperative: Estimate ±SE: 8.97 ± 5.86, *p* = 0.13; HR with 95% CI: 7875.94 (0.08 – Infinity)), previous experience with pulling training (Estimate ±SE: 0.64 ± 1.32, *p* = 0.63; HR with 95% CI: 1.90 (0.14–25.48)) and previous experience with door-opening training (Estimate ±SE: −0.43 ± 1.00, *p* = 0.67; HR with 95% CI: 0.65 (0.09–4.66)). This suggests that the significant effect of past cooperative experience is based on whether or not focal rats were liberated from the captive tube by a partner rather than i) based on the association between the coordinated pulls in the test and experience phases by focal rats and cooperative partners which subsequently had a positive cooperative interaction with them, and ii) the latency until the trapped focal rats became free from the captive tube in the experience phase.

The latency to the first coordinated pull was shorter for focal rats with cooperative partners than for focal rats with non-cooperative partners (Estimate ±SE: 0.91 ± 0.41, *p* = 0.025; HR with 95% CI: 2.50 (1.12–5.55)), which supported our prediction for the direct reciprocity decision rule for coordinated cooperation. The latency to the first coordinated pull was shorter as the mass difference between focal rats and partners increased (Estimate ±SE: 0.013 ± 0.005, *p* = 0.02; HR with 95% CI: 1.01 (1.00–1.02)). The latency to the first coordinated pull tended to be shorter as the number of pulls between the focal rats and the cooperative partners increased in the experience phase (Estimate ±SE: 0.03 ± 0.01, *p* = 0.054; HR with 95% CI: 1.03 (1.00–1.05)). The latency to the first coordinated pull tended to be longer for dyads with previous experience with door-opening training than for dyads without previous experience with door-opening training (Estimate ±SE: −0.74 ± 0.38, *p* = 0.052; HR with 95% CI: 0.48 (0.23–1.01)). The sequence of the first experience phase (non-cooperative vs. cooperative: Estimate ±SE: −0.30 ± 0.36, *p* = 0.41; HR with 95% CI: 0.74 (0.36–1.51)), and previous experience with pulling training (Estimate ±SE: −0.07 ± 0.43, *p* = 0.88; HR with 95% CI: 0.94 (0.40–2.19)) did not influence the latency to the first coordinated pull.

The intervals between coordinated pulls were shorter for focal rats with cooperative partners than for focal rats with non-cooperative partners (Estimate ±SE: 0.33 ± 0.12, *p* = 0.007; HR with 95% CI: 1.39 (1.10–1.77)), which supported our prediction for the direct reciprocity decision rule for coordinated cooperation. The intervals between coordinated pulls were shorter as the number of pulls between the focal rats and the partners increased in the experience phase (Estimate ±SE: 0.008 ± 0.004, *p* = 0.04; HR with 95% CI: 1.01 (1.00–1.02)), and they were also shorter as the mass difference between focal rats and partners increased (Estimate ±SE: 0.004 ± 0.001, *p* = 0.01; HR with 95% CI: 1.00 (1.00–1.01)). The sequence of the first experience phase (Estimate ±SE: −0.21 ± 0.14, *p* = 0.13; HR with 95% CI: 0.81 (0.61–1.07)), previous experience with pulling training (Estimate ±SE: 0.12 ± 0.16, *p* = 0.43; HR with 95% CI: 1.13 (0.83–1.53)) and previous experience with door-opening training (Estimate ±SE: −0.14 ± 0.14, *p* = 0.32; HR with 95% CI: 0.87 (0.66–1.15)) did not influence the intervals between coordinated pulls.

### Kin discrimination and coordinated cooperation

**Figure 3:**
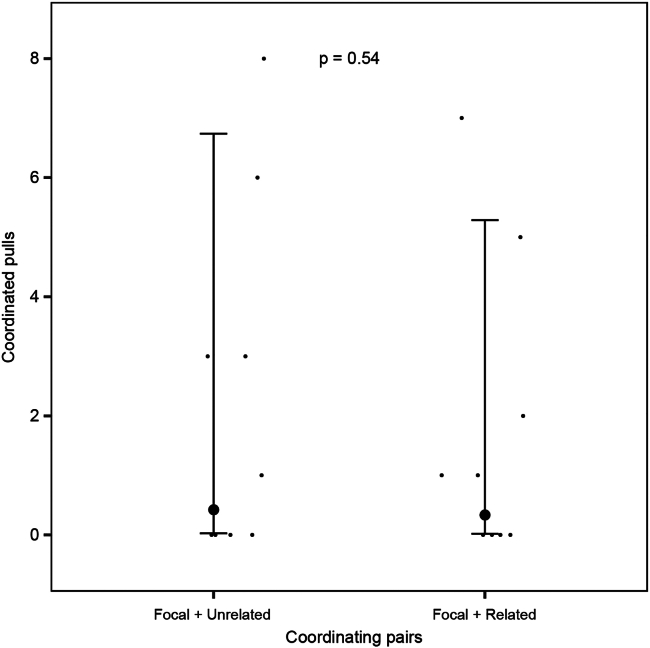
Kin discrimination and coordinated pulling The number of coordinated pulls performed by the coordinating pairs, i.e., focal rats with related partners vs. focal rats with unrelated partners. The large dots with the whiskers represent the mean number of coordinated pulls with 95% confidence intervals, respectively, and the dots represent the raw pulling data. The *p* value for the comparison is equal to 0.54.

The number of coordinated pulls did not differ between focal rats coordinating with related partners and focal rats coordinating with unrelated partners (Estimate ±SE: −0.23 ± 0.37, *p* = 0.54, Figure 3), which did not support our prediction for coordinated cooperation by kin discrimination. The number of coordinated pulls was not influenced by the mass difference between the focal rats and partners (Estimate ±SE: −0.001 ± 0.006, *p* = 0.83), previous experience with pulling training (Estimate ±SE: −0.70 ± 0.86, *p* = 0.41) and previous experience with door-opening training (Estimate ±SE: 0.14 ± 1.37, *p* = 0.92). There was no difference between the full model and the null model without the coordinating pairs, i.e., focal with related partner vs. focal with unrelated partner (Χ^2^ = 0.39, *p* = 0.53).

The latency to the first coordinated pull did not differ between focal rats coordinating with related partners and focal rats coordinating with unrelated partners (Estimate ±SE: 0.45 ± 1.11, *p* = 0.69; HR with 95% CI: 1.57 (0.18–13.87)), which did not support our prediction for coordinated cooperation by kin discrimination. The latency to the first coordinated pull was not influenced by the mass difference between the focal rats and partners (Estimate ±SE: 0.0005 ± 0.0111, *p* = 0.96; HR with 95% CI: 1.00 (0.98–1.02)), previous experience with pulling training (Estimate ±SE: −2.53 ± 2.52, *p* = 0.32; HR with 95% CI: 0.08 (0.00–11.18)), and previous experience with door-opening training (Estimate ±SE: −3.66 ± 2.91, *p* = 0.21; HR with 95% CI: 0.03 (0.00–7.68)).

The intervals between coordinated pulls did not differ between focal rats coordinating with related partners and focal rats coordinating with unrelated partners (Estimate ±SE: −0.03 ± 0.40, *p* = 0.93; HR with 95% CI: 0.97 (0.44–2.10)), which did not support our prediction for coordinated cooperation by kin discrimination. The intervals between coordinated pulls were not influenced by previous experience with pulling training (Estimate ±SE: −0.72 ± 0.47, *p* = 0.13; HR with 95% CI: 0.49 (0.19–1.23)), and previous experience with door-opening training (Estimate ±SE: −17.95 ± 59.36, *p* = 0.76; HR with 95% CI: 1.60 (0.00 – Infinity)).

## Discussion

Our results show that female wild-type Norway rats can coordinate with social partners in a cooperative task. Focal rats cooperated more often, had shorter latencies to the first coordinated action, and had shorter intervals between coordinated actions with cooperative partners that had previously freed them from a confinement tube and subsequently cooperated with them than with non-cooperative partners that had not done so. These results suggest that focal rats recruit more helpful partners to coordinate a cooperative action with them according to the mechanistic decision rule of direct reciprocity, where two different tasks were involved. This is consistent with results from previous experimental studies that found that Norway rats give more and earlier help to cooperative partners than to non-cooperative partners in an iterated prisoner’s dilemma game involving same or different tasks.^62^^,^^63^^,^^64^^,^^65^^,^^66^^,^^67^^,^^68^^,^^69^^,^^83^ In a previous study, focal rats gave more help to lighter partners,^71^ and the effects of the mass difference between focal rats and their partners in our study suggests that Norway rats prefer to recruit lighter partners for cooperative food acquisition, which may help them to succeed in potential competition for the obtained rewards.

The positive effect of coordinated cooperation in the experience phase on the intervals between pulls suggests that focal rats may help based on attitudinal reciprocity or emotion-based reciprocity.^84^^,^^85^ Even though the intervals between pulls in the test phase were shorter with more coordinated cooperation in the experience phase, there was no increase in the number of coordinated pulls given by focal rats in the test phase as the number of coordinated pulls in the experience phase increased. Due to this and other model results our findings suggest that focal rats based their cooperative behavior on whether or not they were liberated by a partner rather than on the association between coordinated pulls in the experience and test phases. A future experimental design might be able to fully distinguish between the past cooperative experience based on whether or not focal rats are liberated by a partner and potential effects of past coordinated cooperation.

Non-cooperative partners in our study could not cooperate, since a locking mechanism prevented the release of the focal rats by the non-cooperative partners. This design for the non-cooperative experience phase provided a past non-cooperative experience for the focal rats, however this design may not reflect the cooperative motivation of the partner. A meta-analysis of reciprocal altruism in Norway rats showed that focal rats reciprocate with partners in an iterated Prisoner’s dilemma based on past cooperative experience according to the direct reciprocity decision rule.^68^ Furthermore, the results from a previous study showed that helpful behavior in rats can be explained by applying direct reciprocity but not by copying tasks performed by social partners through imitation.^69^ Previous studies have shown that direct reciprocity in Norway rats resembles tit-for-tat based on the outcome of the most recent encounter with a specific partner, which was revealed by a series of experience phases with different partners over a time delay between help received and help given of up to 4 days.^86^^,^^87^ The direct reciprocity decision rule can generate evolutionarily stable levels of cooperation^88^^,^^89^ from fitness benefits when pay-offs are correlated by conditional returns, i.e., reciprocity, if there is an above-random chance that an individual’s helpful and costly act increases the likelihood that the individual’s costs of helping will be outweighed by future benefits accrued from receiving help in return.^33^^,^^76^

In a second experiment, focal rats did not cooperatively coordinate more often and sooner with related partners than with unrelated partners. These results are not consistent with focal rats coordinating cooperation according to the kin discrimination mechanism, and they suggest that kinship does not influence how focal rats recruited related and unrelated partners to cooperatively coordinate. This is consistent with a previous study showing that reciprocity but not nepotism explained sequential exchanges of help in Norway rats.^55^ The kin discrimination decision rule can generate evolutionarily stable levels of cooperation from fitness benefits when the pay-offs are correlated by shared genes, i.e., relatedness, which enables kin selection.^33^^,^^76^^,^^78^^,^^89^ Most of the evidence supporting the importance of kin selection is correlational, however.^90^^,^^91^^,^^92^^,^^93^ The results of our experimental study with female Norway rats suggest that the direct fitness benefits of reciprocity among non-kin may be greater than first expected and may even be greater than the indirect fitness benefits.^33^^,^^94^^,^^95^^,^^96^

In conclusion, our experiments reveal that wild-type Norway rats preferably recruit previously helpful partners for a task requiring coordinated cooperation. This suggests that the rats transfer social experience between different tasks, in our case involving rescue behavior and coordinated food acquisition. In contrast, the predicted kin discrimination when cooperatively fetching food was not confirmed. This contrasts with the common belief that relatedness has a stronger influence on cooperative behavior than previous experience with a social partner, i.e., reciprocity,^33^ which highlights the importance of assessing the evolutionarily stable mechanisms underlying coordinated cooperation, such as reciprocal altruism and kin selection. There is an on-going debate over the utility of kin selection and alternative mechanisms.^33^^,^^76^^,^^95^^,^^97^^,^^98^^,^^99^^,^^100^^,^^101^^,^^102^ Our results further inform this debate over the extent to which kin selection and alternative mechanisms may drive altruistic cooperation.

### Limitations of the study

We could not critically assess the influence of the social interaction on some of our results. First, rats liberating conspecifics from the tube were rewarded by obtaining access to food with their partner after pulling in coordination, and potentially also by the social contact per se.^22^^,^^24^^,^^80^^,^^81^^,^^82^ Second, the focal rats had different interaction times with “cooperators” and “non-cooperators” due to the experimental design intended to provide divergent social experience to the focal rats. Third, the intervals between coordinated pulls in the test phase were shorter as the number of coordinated pulls in the experience phase increased, and this result suggests that attitudinal reciprocity or emotion-based reciprocity may play a role. Nonetheless, shorter intervals between pulls did not translate into an increase in the number of coordinated pulls given by focal rats in the test phase as the number of coordinated pulls in the experience phase increased. We ran additional models which showed that past cooperative experience was based on whether or not rats were liberated by a partner rather than on the association between coordinated pulls in the experience and test phases and the latency to liberate the focal rats from the captive tube in the experience phase. Fourth, some dyads were previously paired together during the pulling training or the door-opening training, since both rats paired together in the training and experiments were housed in the same cage. Nevertheless, the intervals were lengthy for rat pairs that were together in both the training and the experiments, and we accounted for the previous experience in the analyses.

## Resource availability

### Lead contact

Requests for further information and resources should be directed to and will be fulfilled by the lead contact, S.C.E. (sacha.engelhardt@uni-goettingen.de).

### Materials availability

This study did not generate new unique reagents.

### Data and code availability

• The datasets have been deposited at Mendeley data and are publicly available as of the date of publication. Accession numbers are listed in the key resources table.
• All original code has been deposited at Mendeley data and is publicly available as of the date of publication. Accession numbers are listed in the key resources table.
• Any additional information required to reanalyze the data reported in this paper is available from the lead contact upon request.

## Acknowledgments

Funding was provided by the 10.13039/501100001711Swiss National Science Foundation (grant number 31003A_176174) to M.T. We thank the animal caretaker, Evi Zwygart.

## Author contributions

N.I.P., S.C.E., and M.T. conceptualized and designed the study. N.I.P. ran the experiments and collected the data from video files. S.C.E. performed the statistical analyses. S.C.E., N.I.P., and M.T. wrote the manuscript and approved the manuscript. M.T. acquired funding and supervised the research project. S.C.E. and N.I.P. are co-first authors. S.C.E., N.I.P., and M.T. agree to be accountable for all aspects, accuracy and integrity of the work.

## Declaration of interests

The authors declare no competing interests.

## STAR★Methods

### Key resources table

**Table:** 

REAGENT or RESOURCE	SOURCE	IDENTIFIER
**Experimental models: Organisms/strains**		

Outbred, wild-type Norway rats (*Rattus norvegicus*; 63 females)	Animal Physiology Department, University of Groningen, Netherlands	N/A

**Deposited data**		

Data files	This paper	https://doi.org/10.17632/hhzttn5pmy.2

**Software and algorithms**		

R: A Language and Environment for Statistical Computing	R Core Team^103^	https://www.R-project.org/
Original code	This paper	https://doi.org/10.17632/hhzttn5pmy.2
lme4	Bates et al.^104^	https://doi.org/10.18637/jss.v067.i01
lmerTest	Kuznetsova et al.^105^	https://doi.org/10.18637/jss.v082.i13
survival	Therneau^106^; Therneau and Grambsch^107^	https://CRAN.R-project.org/package=survival
coxme	Therneau^108^	https://CRAN.R-project.org/package=coxme
frailtypack	Rondeau et al.^109^; Rondeau and Gonzalez^110^	https://doi.org/10.18637/jss.v047.i04 https://doi.org/10.1016/j.cmpb.2005.06.010
effects	Fox and Weisberg^111^^,^^112^	https://socialsciences.mcmaster.ca/jfox/Books/Companion/index.html https://doi.org/10.18637/jss.v087.i09
ggplot2	Wickham^113^	https://ggplot2.tidyverse.org
car	Fox and Weisberg^111^	https://socialsciences.mcmaster.ca/jfox/Books/Companion/
ggcorrplot	Kassambara^114^	https://CRAN.R-project.org/package=ggcorrplot
DHARMa	Hartig^115^	https://CRAN.R-project.org/package=DHARMa
fitdistrplus	Delignette-Muller and Dutang^116^	https://cran.r-project.org/web/packages/fitdistrplus/index.html
glmmTMB	Brooks et al.^117^	

### Experimental model and study participant details

#### Subjects and holding conditions

Sixty-three female, outbred, wild-type Norway rats, *Rattus norvegicus*, (source: Animal Physiology Department, University of Groningen, Netherlands) were used. We drove the rats to the Ethologische Station Hasli of the University of Bern, Switzerland, where the study took place. To visually distinguish individuals, upon arrival the rats were marked by ear punches, which caused light momentary bleeding at the ear. If blood was visible after the ear-punching procedure, we stopped the bleeding by gently pressing on the ear with a paper tissue for 10 s, after which the bleeding stopped. At the start of the experiments, the mean mass of rats was 298.1 g ± 3.8 g, and the mean age of rats was 502.2 days ± 4.1 d. The rats were habituated to handling from weaning onwards and did not show signs of stress during rearing and all experimental stages. Rats were handled regularly to keep them habituated to the experimenters. The average ambient temperature was 20 ± 1°C, and the relative humidity ranged from 50% to 60%. The rats were kept in 13 sister groups of four or five rats each. Home cages (80 cm × 50 cm x 37.5 cm) contained a wooden house, a wooden platform and a wooden cylinder as well as a plastic tunnel, wood shavings, and hay for nesting material. Home cages were separated from each other by opaque walls to limit interactions between groups. Conventional rat pellets and water were accessible *ad libitum* (except when temporary fasting was applied for the experiments, see the sub-section “experimental tests” for details), and fresh food (fruits and vegetables) and seed mix were supplemented twice a week and four times a week, respectively. As rats are nocturnal we employed an inversed 12:12 h light:dark cycle with a 30min dawn/dusk period of dim lighting. Lights began to dim at 08:00 h and were completely off at 08:30 h to allow us to work during their active period. Red lights were used during all experimental procedures to enable the observation of the rats during dark hours without affecting the animals, as they possess a low sensitivity toward red light.^118^ We performed daily health checks.

#### Ethics statement

The license to perform animal experiments was provided by Swiss Federal Veterinary Office of the Canton of Bern (license number BE 55/18), which was co-authored by S.C.E. and M.T. A ticket for indispensable research was provided by the University of Bern (ticket number EAC-201216-T#212) to M.T.

### Method details

#### Experimental setup and apparatus

The experimental setup was based on a modified version of an established food-sharing task where Norway rats pull a stick attached to a moveable tray to provide a partner, but not itself, with food.^59^^,^^62^^,^^66^^,^^86^ The experimental apparatus is a modified version of the cooperative pulling paradigm,^119^ which can only be successfully operated when two or more individuals coordinately pull a panel toward themselves to gain access to food rewards.^37^^,^^47^^,^^120^ We designed a tray with a protruding food well located between two sticks spaced 5cm apart. We used sticks as handles as previous studies have shown that Norway rats are prone to pull sticks to access food rewards for themselves (solo pulling task) and for partners (social pulling task).^59^^,^^62^^,^^66^^,^^67^^,^^86^ The tray was placed at the center of the long side of an experimental cage (80 cm × 50 cm x 37.5 cm) with a hole cut into it to allow the tray and sticks to slide inside without allowing the rats to exit the cage (Figure 1). Screws connected to wingnuts were attached in such a way that they pushed down on a plastic sheet at the base of the platform to allow for adjustment of the force required to move the tray by increased friction (hereafter referred to as resistance).

#### Pre-experimental training

##### Pulling training

Sixty-three Norway rats were trained to pull a stick attached to a moveable tray to obtain food (oats) for themselves over the course of seven training sessions. The rats could acquire the food by pulling a single stick with their teeth so that the tray entered through a hole in the side of the cage. We considered the task to have been properly learned after each rat had successfully pulled food for itself at least 10 times per training session for three consecutive training sessions. After seven training sessions, each rat had successfully pulled food for itself between 27 and 130 times (median: 88) for the last three sessions, and all individuals were moved to the next stage of training.

In the second phase of training, a second stick was introduced on the other side of the food well of the moving tray in a procedure designed to train the rats to acquire food by pulling jointly at the same time. All rats were paired up with another individual from their home cage. Once inside the training cage, a dyad of rats would initially only receive a food reward when both individuals were touching one, or both, of the two sticks, and then only after both rats pulled the sticks at the same time, with solo pulling being prevented by the experimenter. Following nine training sessions using this blocking technique, we added a resistance to the tray at 100 g increments from 500 g to 900 g after each successful coordinated pull, which allowed rats to pull by themselves, but the task could be made easier by recruiting a partner. During this training we recorded all attempts at solo pulls and all coordinated pulls made by the rats.

#### Door-opening training

All rats were habituated to being restrained within a transparent Plexiglas confinement tube (inner diameter: 15cm, length: 27cm; see Figure S1) with an opaque door and back, both of which had been perforated to allow the transfer of olfactory and auditory information. Thereafter, all focal rats and cooperative partners were trained to open the door of the confinement tube by pulling a horizontal stick, causing the door to fall open on vertically oriented hinges. In a first step of training, all rats were taught to open an empty tube by the administration of a food reward (oats) upon successful opening of the door. Once all rats would open the door a minimum of five times in a 10-min session, a partner from the same home cage as the opening rat was placed inside the tube. Again, all rats received a treat when opening the door, and they could interact with the liberated partner.^22^^,^^24^^,^^80^^,^^81^^,^^82^ Over 20 min training sessions, the roles of opener and restrained rats were interchanged each time the door was opened until each rat opened the door at least five times in a session. This alternate turn-taking of the role as opener and restrained rat went on for 20 min, for as many sessions as was required until both partners had opened at least five times (total of ten openings in a session; number of sessions: mean ± SE = 4.95 ± 0.23, range = 4–7 sessions).

#### Experimental tests

Each session of both experimental tests of coordinated cooperation involved one focal rat and two partners from the same home cage for a total of 54 individuals. Each of the 54 individuals passed the training sessions. The decision to use cage mates was based on an earlier pilot study that revealed that despite being offered the opportunity to work together to acquire a food reward, rats from different home cages would rather engage in low-intensity agonistic interactions than coordinate pulling to acquire food. All tests occurred in a test cage of the same dimensions as a home cage, and a section of the bars at the center of the long side of the cage had been removed to allow the tray and pulling sticks to be inserted. To increase the motivation of rats to coordinate pulling to acquire food in the experiments, all rats had all food removed from their home cage in the afternoon of the day prior to testing, and food was returned after testing (water was available *ad libitum* during the food removal period). For this reason, no cage was used for testing on two consecutive days to minimize prolonged hunger periods.

#### Experiment 1: Direct reciprocity

To assess the direct reciprocity decision rule for coordinated cooperation, we ran an experiment utilizing a moving tray with resistance to test whether female Norway rats would rather coordinate efforts to solve a cooperative task with a previously cooperative partner relative to a partner that had previously not done so, i.e., a non-cooperative partner. In this study, we used 19 focal rats, and a total of 20 partner rats (10 cooperative partners and 10 non-cooperative partners). Focal and partner rat roles were not reversed, such that focal rats were never partners and partners were never focal rats. Of the 38 experimental dyads (19 dyads each consisting of a focal rat with a cooperative partner and 19 dyads consisting of a focal rat with a non-cooperative partner), 26 (68.4%; 13 dyads composed of focal rats and cooperative partners and 13 dyads composed of focal rats and non-cooperative partners) dyads included rats that had been previously paired together during the pulling training, but this had occurred a long time before the experiment (mean ± SE: 128.9 ± 11.3 days, median: 165.5 days, range: 48–207 days). Sixteen (42.1%; 8 dyads composed of focal rats and cooperative partners and 8 dyads composed of focal rats and non-cooperative partners) of the 38 dyads included rats that had been previously paired together during the door-opening training, which again had occurred a long time before the experiment (mean ± SE: 84.9 ± 0.9 days, median: 85.5 days, range: 80–90 days). We accounted for these lengthy intervals between training and the experiment in the analyses. Each focal rat first encountered the two partners separately in two experience phases (Figure 1A) in a balanced random order with a 2-h delay between the two experiences. Each experience phase started with the pulling sticks connected to the feeding tray inserted into the cage, the focal rat inside a transparent confinement tube, and a partner in the test cage. Each of the 20 partner rats were involved in only 1 experience phase and 1 test phase per day with 1 focal rat. All cooperative partners had been trained to open the confinement tube. If cooperative partners did not open the confinement tube within 5 min after the start of the experience phase, i.e., after being introduced into the cage, the door was opened by the experimenter to allow the freeing of the focal rat. As social contact following the freeing of a partner seems to be rewarding to rats,^22^^,^^24^^,^^80^^,^^81^^,^^82^ we used it to incentivize the focal rats to help the cooperative partner acquire food rewards via coordinated pulling, thereby establishing a cooperative experience for the focal rat. Prior to the freeing of the focal rat, the platform was locked in place. Following the freeing of the focal rat, the platform was unlocked for 10 min, and the two rats could pull the food-baited tray set at a resistance of 800 g to access food rewards (oats) for each coordinated pull. In contrast, non-cooperative partners could not open the confinement tube, and a locking mechanism that could not be opened was in place to avoid accidental openings. After a 150s delay following the introduction of the non-cooperative partner, the sticks were inserted to allow the non-cooperative partner to acquire food for itself for 10 min with the tray resistance lowered to 600g to make it easier for a single rat to move the tray unassisted to access food rewards (1 oat per pull) (duration of the experience phase with a non-cooperative partner: 12.5 min). The use of a transparent and perforated confinement tube meant the focal rat could receive both the limited visual information allowed by the red light illumination as well as auditory and olfactory information from the partners, e.g., the non-cooperative partners pulling and eating food rewards.

Three hours after the second experience phase, the focal rat and both partners were brought back to the test cage for the test phase. There was no confinement tube during the test phase, and all three rats could interact freely throughout the test phase (Figure 1A). A moving tray was installed in front of the cage, and the moving tray glided with ball bearings on two rails. The moving tray was modified from previous cooperation experiments with Norway rats.^62^^,^^66^^,^^71^ Two sticks were attached to the moving tray so that it could be reached by the rats in the cage (Figure S2). Once all rats had been placed inside the test cage two sticks connected to the moving tray were inserted at the center of the long side of the test cage, allowing the three rats to freely pull together with either of, or both of, the two conspecifics. The rats pulling jointly (in the cooperative experience phase and in the test phase) or alone (in the non-cooperative experience phase) moved the tray closer to the cage, so the food rewards on the tray could be accessed. The resistance of the moving tray was increased manually with the help of adjustable screws. In the test phase, the resistance was set to 800 g. All attempts by a single rat at pulling the platform were blocked by the experimenter. After each successful pull in both experience and test phases, rats could eat the food rewards. Each test phase lasted for 10 min, after which the sticks were removed followed by the return of all rats to their home cage.

#### Experiment 2: Kin discrimination

This experiment tested whether female Norway rats differentially solve a cooperative coordination task with a full sister (related) than with a conspecific with which they shared no parents (unrelated). Nine rats were chosen as focal rats, and 13 rats were chosen as related and unrelated partners with some individuals acting as the related partner to one focal and the unrelated to another, depending on composition of rat groups in the holding cages. Of the 18 dyads, 8 (44.4%; 5 dyads composed of focal rats and related partners and 3 dyads composed of focal rats and unrelated partners) included rats that had been previously paired together during the pulling training, but this had happened a long time before the experiment (mean ± SE: 137.5 ± 21.6 days, median: 161.0 days, range: 67–206 days). One (5.6%; 1 dyad composed of the focal rat and a related partner) of the 18 dyads included rats that had been previously paired together during the door-opening training, which had occurred 79 days before the experiment. We accounted for these lengthy intervals between training and the experiment in the analyses. A focal rat was placed in a test cage with a related and unrelated partner (Figure 1B), and two sticks connected to the moving tray were inserted at the center of the long side of the test cage, allowing the three rats to freely coordinate pulls. Resistance to access food rewards was set to 800 g, and the experimenter blocked solo pulling attempts. Rats could access food rewards (oats) after each coordinated pull during 10 min, after which the rats were returned to their home cage.

#### Behavioral data

All observations were video-recorded using a handycam with night-vision mode (Sony HDR-CX550) and analyzed at the end of the experiment. We recorded the number of coordinated pulls and the delay to each coordinated pull by the focal rats and their partners. All interactions were monitored for agonistic interactions. Only 1 agonistic interaction was observed, i.e., a rat was mounted by its partner in one of the sessions of the test phase of the direct reciprocity decision rule experiment. Thus, agonistic interactions were not considered for the final analysis.

### Quantification and statistical analysis

All means and coefficients are reported with standard errors or 95% confidence intervals (CI), and an alpha of 0.05 was chosen. Statistical analyses were performed using R^103^ and the packages “lme4”,^104^ “lmerTest”,^105^ “survival”,^106^^,^^107^ “coxme”,^108^ “frailtypack”,^109^^,^^110^ “effects”,^111^^,^^112^ “ggplot2”,^113^ “ggcorrplot”,^114^ “DHARMa”,^115^ “fitdistrplus”^116^ and “glmmTMB”^117^ packages.

#### Solo and coordinated pulling training

During the training phase experimental subjects learnt first to pull a tray toward their cage in order to collect food deposited on it, before they could only obtain food if a cage mate joined their effort. In the second pulling training phase the rats could decide to pull on their own or with the help of their cage mate, while the resistance of the pulling mechanism was progressively raised. To validate that the number of solo pulls decreased with increasing number of training sessions in the second pulling training phase, we ran a generalized linear mixed model with a zero-inflated negative binomial distribution with the number of solo pulls as the response variable, the number of training sessions as a fixed effect with dyads as the random intercept effect. The number of solo pulls decreased as the number of sessions increased in this second pulling training phase (Estimate ±SE: −0.16 ± 0.01, *p* < 0.001), which validated the training effect. The comparison between the full model and the null model without the number of training sessions, i.e., the intercept-only model, revealed that the number of training sessions significantly affected the number of solo pulls (Χ^2^ = 241.9, *p* < 0.001).

In a previous study, the number of pulls by focal rats decreased as the resistance increased.^71^ We expected an initial increase in coordinated pulls as the number of training sessions increased, but the coordinated pulls should then decrease with progressing training sessions due to the gradual increase in resistance from 500 g to 900 g. We ran a generalized linear mixed model with a zero-inflated negative binomial distribution with the number of coordinated pulls as a response variable, the number of training sessions (z-scored) and the number of training sessions squared (z-scored) as a quadratic term as fixed effects with dyads as the random intercept effect. When the number of sessions (z-scored) was equal to zero, the number of coordinated pulls would increase by 0.28 for every additional training session (Estimate ±SE: 0.28 ± 0.04, *p* < 0.001), if the slope remained unchanged but it was quadratic. Each additional session reduced the slope by 0.49 (Estimate ±SE: −0.49 ± 0.04, *p* < 0.001). Since the quadratic estimate for the number of sessions was negative, the relationship was concave. There was an initial increase in coordinated pulls with more training sessions, but the gain in coordinated pulls decreased with additional training sessions. The decreasing trend with additional training sessions was apparently due to the increased resistance. The comparison between the full model and the null model without the number of training sessions, i.e., the intercept-only model, revealed that the number of training sessions significantly affected the number of coordinated pulls (Χ^2^ = 156.12, *p* < 0.001).

#### Direct reciprocity: Coordinated cooperation

We ran a generalized linear mixed model (GLMM) with a negative binomial distribution to account for overdispersion of residuals, i.e., the residuals for a GLMM with a Poisson distribution were overdispersed, with the number of coordinated pulls in the test phases as a response variable. When the proportional hazard assumption was met, we ran semi-parametric event history analyses, however we ran parametric event history analyses when this assumption was not met. We ran a mixed effects parametric event history analysis with a Weibull distribution with the latency to the first coordinated pull as the response variable. We ran a mixed effects parametric event history analysis with a Weibull distribution with the intervals between coordinated pulls as the response variable. For each of these models, the coordinating pairs (i.e., categorical variable with 2 levels: focal rat and cooperative partner, and focal rat and non-cooperative partner as the level of comparison) was the main fixed effect. We included the number of pulls with the partner in the experience phase, the mass difference between focal rats and partners, the first experience phase (cooperative or non-cooperative) to account for any sequence effect of the experience phases as fixed effects. We included whether or not dyads had previous experience during the pulling training and during the door-opening training to account for dyads with previous experience as fixed effects. For the number of coordinated pulls, the identities of the focal rats, cooperative partner rats and non-cooperative partner rats were included in the test phase as random intercept effects. For the latency to the liberation of the focal rats from the captive tube, the identities of the focal rats were included as a random intercept effect, which accounted for repeated observations. For the intervals between coordinated pulls model, a single factor combining the focal rats and the experience phase (cooperative or non-cooperative) was used as a random intercept effect, which accounted for repeated observations.

We ran two additional models to assess if the effect of past cooperative experience is based on i) the association between the number of coordinated pulls in the test and experience phases between only the focal rats and the cooperative partners, or ii) the latency to liberate focal rats from the captive tube. To assess if the association between coordinated pulls in the experience phase between the focal rats and the cooperative partners affected how often focal rats pulled with the cooperative partners in the test phase, we ran a GLMM with a Poisson distribution with the number of coordinated pulls in the test phase between the focal rats and the cooperative partners as the response variable. We included the number of coordinated pulls with the cooperative partner in the experience phase (the main fixed effect), the mass difference between focal rats and cooperative partners, the first experience phase (cooperative or non-cooperative) to account for any sequence effect of the experience phases, and whether or not dyads had previous experience during the pulling training and during the door-opening training to account for dyads with previous experience as fixed effects. To assess if the latency to liberate the focal rats from the captive tube by partners affected how often focal rats coordinated with partners, we ran a parametric event history analysis with a Weibull distribution for the latency to liberate the focal rats from the captive tube with the experience phase (i.e., categorical variable with 2 levels: cooperative and non-cooperative, as the level of comparison) as the main fixed effect. For partners that liberated the focal rats, the latency to liberate the focal rats was set to the latency to liberate and success was set to 1. For partners that did not liberate the focal rats, the latency to liberate the focal rats was set to 300 s and the success to liberate was set to 0. We also included whether or not dyads had previous experience during the pulling training and during the door-opening training to account for dyads with previous experience as fixed effects. We ran an ANOVA to compare the full model to the null model without the main fixed effect of interest, however we could not compare the full model to the null model for the parametric event history analyses.

#### Kin discrimination: Coordinated cooperation

We ran a generalized linear mixed model (GLMM) with a Poisson distribution with the number of coordinated pulls as a response variable. We ran a mixed effects parametric event history analysis with a Weibull distribution with the latency to the first coordinated pull as the response variable. We ran a mixed effects parametric event history analysis with a Weibull distribution with the intervals between coordinated pulls as the response variable. For each of these models, the coordinating pairs (i.e., categorical variable with 2 levels: focal rat and related partner, and focal rat and unrelated partner as the level of comparison) was the main fixed effect. We included whether or not dyads had previous experience during the pulling training and during the door-opening training to account for dyads with previous experience as fixed effects. We included the mass difference between focal rats and partners as a fixed effect in the number of coordinated pulls model and in the latency to the first coordinated pull model. There was only one dyad with previous experience with door-opening training, and only one interval between coordinated pulls: we kept the variable in the analysis. For the number of coordinated pulls model, the identities of the focal rats, the related partners and the unrelated partners were included as random intercept effects. For the latency to the first coordinated pull and the intervals between coordinated pulls models, the identity of focal rats was included as a random intercept effect. We ran an ANOVA to compare the full model to the null model without the main fixed effect of interest, however we could not compare the full model to the null model for the parametric event history analyses.
