# Supplementary material for: Norway rats recruit cooperation partners based on previous receipt of help while disregarding kinship
Source: iScience. 2024 Nov 4;27(12):111314. doi: 10.1016/j.isci.2024.111314 (PMC11638632; doi:10.1016/j.isci.2024.111314)

iScience, Volume *27*

Supplemental information

Norway rats recruit cooperation partners based on previous receipt of help while disregarding kinship

Sacha C. Engelhardt, Niklas I. Paulsson, and Michael Taborsky

Supplemental information

Figure S1 The confinement tube used in the direct reciprocity decision rule and coordinated pulling experiment.

Focal rats and cooperative partners were trained to open the door of the confinement tube by pulling a horizontal stick, causing the door to fall open on vertically oriented hinges. All rats were habituated to being restrained within a transparent Plexiglas confinement tube (inner diameter: 15cm, length: 27cm) with an opaque door and back, both of which had been perforated to allow the transfer of olfactory and auditory information to pass unhindered.

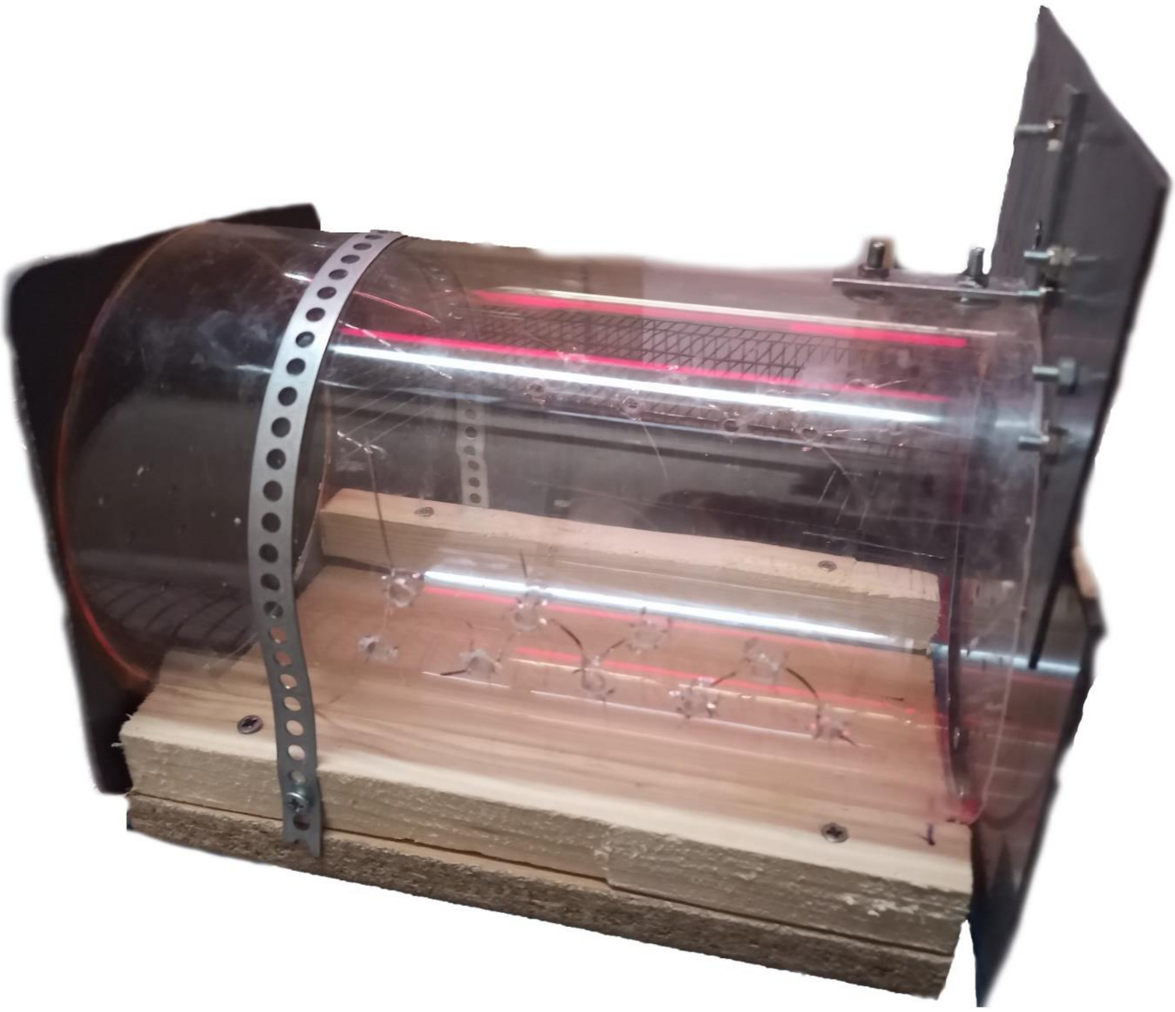

Figure S2 A moving tray, T, was installed in front of the cage, C, and the moving tray glided with ball bearings on two rails. Two sticks, S, were attached to the moving tray so that it could be reached by the rats in the cage. Once all rats had been placed inside the test cage two sticks connected to the moving tray were inserted at the center of the long side of the test cage, allowing the three rats to freely pull together with either of, or both of, the two conspecifics. The rats pulling jointly (in the cooperative experience phase and in the test phase) or alone (in the non-cooperative experience phase) moved the tray closer to the cage, so the food rewards, R, on the tray could be accessed. The resistance of the moving tray was increased manually with the help of adjustable screws.

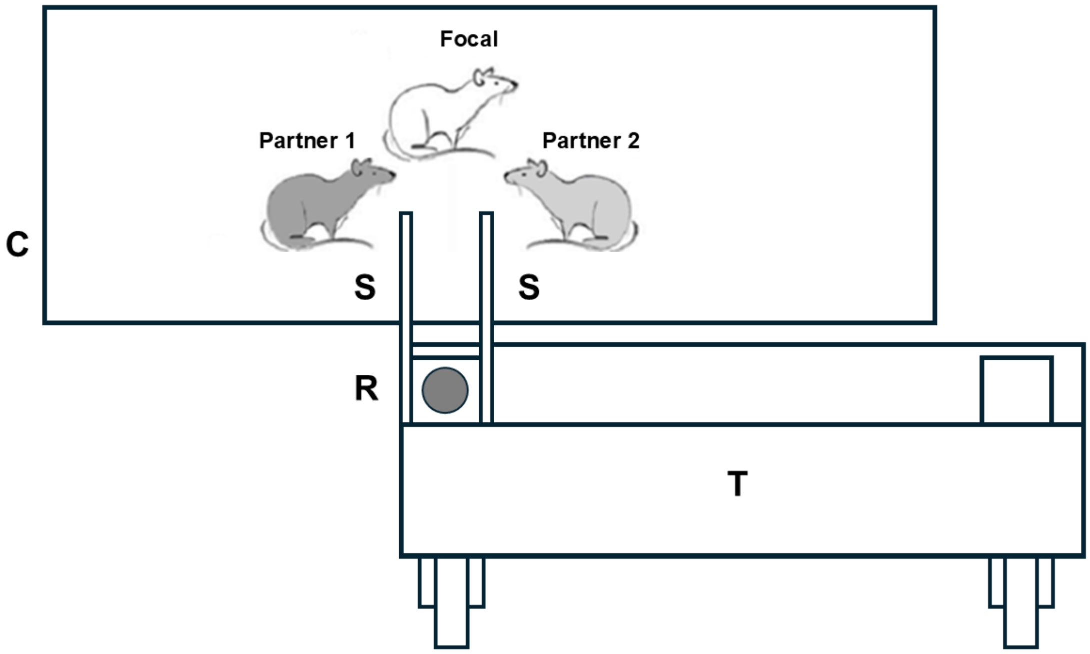

Supplement: Document S1. Figures S1 and S2 [file mmc1.pdf]
